# SAIC: an iterative clustering approach for analysis of single cell RNA-seq data

**DOI:** 10.1186/s12864-017-4019-5

**Published:** 2017-10-03

**Authors:** Lu Yang, Jiancheng Liu, Qiang Lu, Arthur D. Riggs, Xiwei Wu

**Affiliations:** 1 Integrative Genomics Core, Beckman Research Institute, City of Hope, Duarte, CA 91010 USA; 2 Department of Developmental and Stem Cell Biology, Beckman Research Institute, City of Hope, Duarte, CA 91010 USA; 3 Diabetes and Metabolism Research Institute, City of Hope, Duarte, CA 91010 USA; 4 Department of Molecular and Cellular Biology, Beckman Research Institute, City of Hope, Duarte, CA 91010 USA

**Keywords:** Single cell, RNA-seq, Clustering, K-means, ANOVA, PCA, Signature genes, T-SNE

## Abstract

**Background:**

Research interests toward single cell analysis have greatly increased in basic, translational and clinical research areas recently, as advances in whole-transcriptome amplification technique allow scientists to get accurate sequencing result at single cell level. An important step in the single-cell transcriptome analysis is to identify distinct cell groups that have different gene expression patterns. Currently there are limited bioinformatics approaches available for single-cell RNA-seq analysis. Many studies rely on principal component analysis (PCA) with arbitrary parameters to identify the genes that will be used to cluster the single cells.

**Results:**

We have developed a novel algorithm, called SAIC (Single cell Analysis via Iterative Clustering), that identifies the optimal set of signature genes to separate single cells into distinct groups. Our method utilizes an iterative clustering approach to perform an exhaustive search for the best parameters within the search space, which is defined by a number of initial centers and *P* values. The end point is identification of a signature gene set that gives the best separation of the cell clusters. Using a simulated data set, we showed that SAIC can successfully identify the pre-defined signature gene sets that can correctly separated the cells into predefined clusters. We applied SAIC to two published single cell RNA-seq datasets. For both datasets, SAIC was able to identify a subset of signature genes that can cluster the single cells into groups that are consistent with the published results. The signature genes identified by SAIC resulted in better clusters of cells based on DB index score, and many genes also showed tissue specific expression.

**Conclusions:**

In summary, we have developed an efficient algorithm to identify the optimal subset of genes that separate single cells into distinct clusters based on their expression patterns. We have shown that it performs better than PCA method using published single cell RNA-seq datasets.

**Electronic supplementary material:**

The online version of this article (doi:10.1186/s12864-017-4019-5) contains supplementary material, which is available to authorized users.

## Background

Most molecular biology studies in the past decades have been based on the data of gene expression levels over an entire population of cells, assuming that characteristics of these cells are homogenous. However, recent single cell studies have proved this assumption to be incorrect, as cell-to-cell variation even exists in genetically identical cells [1, 2]. Such heterogeneities among individual cells may decide different cell-fate in response to environmental stress [3, 4] or biological conditions [5]. Fortunately recent technology advances, especially in the field of microfluidics, enables massively parallel isolation and preparation of individual cells for large-scale whole transcriptome studies to survey heterogeneity and discover novel cell populations [6]. Although there are well-established bioinformatics approaches for analyzing bulk-cells gene expression data, there are still limited analysis approaches for single cell data studies due to the complex and heterogynous nature of single cell gene expression data. Consequently there are increasing interests to develop bioinformatics methods to address the issues of normalization, gene expression signature genes searching, sub-population identification, and clustering in single cell RNA-seq data analysis.

One of the most common goals of single cell study is to identify sub-populations of the cells under certain biological condition. Thus finding the most useful subsets of genes, whose expression patterns would help in clustering the single cells, becomes the key step of the entire analysis workflow. Many current single cell data analysis approaches focused only on the clustering algorithm but were not engaged in searching for signature genes that can benefit the clustering step. These methods are conducted on genes filtered by RPKM [7, 8] values or the top genes that have the largest residuals after fitting a simple noise model [9]. Therefore the number of the genes used for clustering can be as large as thousands. At this scale, clustering results may be affected or even driven by the noise embedded in gene expression data. For downstream analysis, such as biological validation and marker genes selection, it would be very difficult to study a large number of genes. Therefore, it will be ideal if a smaller subset of genes can be selected and are capable of clustering the cells into distinct groups.

Some studies rely on traditional PCA methods [10–12] to identify a set of representative genes, which are then used to further separate single cells into different clusters. For example, in Treutlein et al.’s recent report on single cell analysis of lung cells [11], 28 genes with highest loadings of the first four principle components were used in unsupervised clustering. The parameter selection for PCA-based signature genes selection approach is rather arbitrary, such as which principle components to use and the number of genes with highest loadings. Macro et al. recommended using the 1000 most variable genes [10]. Seurat, an R toolkit, combines linear and non-linear dimensionality reduction algorithms for unsupervised clustering of single cells [13]. Seurat also relies on PCA to select a set of highly variable genes to be used in downstream clustering steps. Exact parameter settings for this step vary empirically from dataset to dataset. Another single cell analysis tool ICGS (https://code.google.com/p/altanalyze/wiki/ICGS) uses the Iterative Clustering and Guide-gene Selection algorithm to identify the most coherent, correlated gene signatures that are able to provide a better single cell clustering in the later step. A key parameter of this method, the correlation threshold, must be provided to indicate the minimum relative similarity required to report correlated genes for downstream analyses, which is also empirical on different datasets. In general, these approaches all require arbitrary or empirical parameters to conduct the signature gene selection procedure.

In this study, we developed an iterative bioinformatics approach that can identify the subset of signature genes whose expression patterns can reliably cluster the single cells into distinct groups. The initial parameter setting of our method is minimally empirical and less arbitrary than PCA based methods.

## Methods

### Data sources

In this study we used three datasets including two published single cell datasets [11, 14] and one simulated data.

1. Lung epithelial cells dataset: Eighty single embryonic lung epithelial cells were captured and mRNAs were sequenced. Gene expression levels were quantified as fragments per million mapped reads (FPKM) generated by TopHat/Cufflinks. [15]
2. Cell mixture dataset: 301 single cells were captured from a mixture of 11 cell populations and mRNA-seq was performed. Quantification of gene expression levels were represented as TPM (transcripts per million) using RSEM v1.2.4. [16]
3. We simulated a dataset containing 5000 genes and 100 samples. A subset of genes (about 5% of total genes) had deviated expression profiles than the rest of genes, which can separate the samples into 10 clusters. Each cluster contained 10 samples and had various numbers of up and down-regulated genes.

### Data preprocessing

To prepare the data for the signature genes selection step, we used the following filters:

1. Removed all the genes expressed below a cutoff (FPKM > =1 in lung dataset and TPM > =1 for cell mixture dataset) in less than 2% of the cells to remove undetectable genes.
2. For the remaining genes, we calculated the mean and coefficient of variation (CV; equals standard deviation divided by the mean) for each gene. We fitted a loess model using (log2 transformed) CV and mean values in R and then chose all the genes with a distance more than 0.1 above the fitted line for further signature genes selection [9].

### Signature genes selection using iterative clustering

Our method utilizes an iterative k-means [17] clustering approach to perform an exhaustive search for the best signature genes within the search space, which is defined by the combination of a number of initial centers K and *p*-values P. The iteration is an optimization process (evaluated by Davies-Bouldin index [18]) for each parameter combination to select the best signature genes given the predefined number of cluster *k* and significant *p* value.

Minimize:

*DB* = *f*(*k*, *p*)

Subject to,

$$ k\in K,p\in P $$

**Fig. 1:**
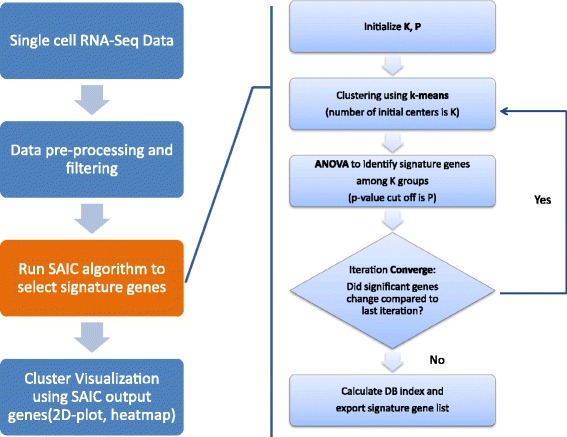
Systemic scheme of SAIC algorithm

As the schematic workflow shown in Fig. 1, for each number of initial centers (*k*) and *p*-value (*p*) combination, a k-means clustering using *k* as the initial number of centers is performed on gene expression matrix (log2 transformed FPKM or TPM) and analysis of variance (ANOVA) is then used to analyze the differences of gene expression values among k groups for each gene. Genes with ANOVA calculated *p*-value less than or equal to the preset *p* are entered into the next round of k-means clustering using the same *k* as initial number of centers. The iteration continues until the number of genes after the iteration remains unchanged from the previous iteration. We consider that the optimal gene subset’ is stable for this parameter combination. At the end of iteration, a Davies-Bouldin (DB) index will be calculated for each parameter combination based on the selected signature genes and k-means determined clusters. DB index, with the formula shown below, is a commonly used scoring function to evaluate the clustering result. S_i_ is a measure of scatter within the cluster i; d (C_i_, C_j_) is a measure of separation between cluster ci and cj. It is a function of the ratio between the within cluster scatter and the between cluster separation, therefore a lower DB index indicates a better clustering.

$$ DB=\frac{1}{N}\sum_{i=1}^N\underset{i\ne j}{\max}\left(\frac{S_i+{S}_j}{d\left({C}_{i,}{C}_j\right)}\right) $$

The best combination of cluster number and *p*-value will be selected according to DB index values. Gene sets associated with this best combination will be considered as the optimal signature gene sets.

### Visualization of clustering using signature genes

For visualizing the clustering results of single cell data, we adopted R toolkit Seurat [13], which combines linear dimension-reduction method (PCA) and nonlinear dimension-reduction method (t-SNE). Instead of applying the combined dimension-reduction to variable genes identified by Seurat, we used the signature genes selected from our iteration process as input to Seurat. Density clustering then was used to classify distinct groups of cells on the t-SNE map created from these genes. The signature genes were also subjected to unsupervised hierarchical clustering using Cluster v3.0 [19] and visualized using Java Treeview [20].

### Implementation and hardware environment

The entire algorithm was implemented in R. The computation server used to run the algorithm was a DELL server with 32 Intel Xeon CPU cores and 256G RAM.

## Results

We applied the SAIC algorithm to one simulated dataset and two published single cell datasets. After signature genes selection, the results were evaluated by Davies-Bouldins index and then visualized using both a t-SNE 2D–plot and an unsupervised hierarchical clustering heatmap.

### Simulated data set

**Fig. 2:**
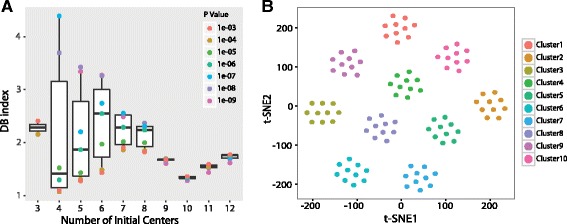
SAIC performance on the simulated dataset. **a** Distribution of DB index values for each initial center using SAIC algorithm on the simulated data set. Boxplot was generated using R package “ggplot”. Each box represents a range of *p* values for the specific initial center parameter (K). Each dot represents the actual DB index value of each *p*-value (P). The middle line within each box represents the median value. Whiskers stop at lower and upper adjacent values. **b** Two-dimensional plot (t-SNE) of the 100-cell simulated data. The 619 signature genes were selected using SAIC algorithm. Clusters are colored by predefined groups

We applied the SAIC algorithm to the simulated dataset consisting of 100 cells with signature genes that can separate the cells into 10 clusters. The first step was to select a reasonable search space, defined by a range of K and *P* values. We selected K ranging from 3 to12, which allowed us to evaluate the effects of sub-optimal cluster numbers. We selected *P* values ranging from 0.001 to 1e-09 as our search space. We applied the SAIC algorithm with these combinations, and the distribution of DB index values is shown in Fig. 2a. The median DB index for K = 3 is 2.13. It is interesting that the DB index decreases when the initial center becomes closer to the correct number of 10, but increases again when the initial center number exceeded 10. Large variation in the DB index can be observed when the initial center number is small, while this variation reduces as the initial center approaches 10. The DB indexes also become smaller as the *p* value became more stringent, and results in less signature genes. The results show that an initial center of 10 gives the best overall DB index, while the optimum parameter combination is K = 10 and *P* = 1e-09 (Fig. 2a). This parameter combination resulted in 619 signature genes. Two-dimensional t-SNE plot shows that these signature genes can separate the 100 cells correctly into the 10 pre-defined clusters (Fig. 2b). We also performed analysis using PCA and the Seurat method. For PCA method, we combined the top 50 genes of the first 4 principal components to select 347 unique genes. For Seurat, we picked the first 3 principal component and significant level of 0.01, which resulted in 183 unique genes. Neither of the methods is able to identify the 10 subgroups correctly (Additional file 1: Figure S1).

Although the algorithm worked well with simulated data, it is important to apply it to experimentally generated single cell data sets to prove its effectiveness for real biological data.

### Lung epithelial cells data

**Fig. 3:**
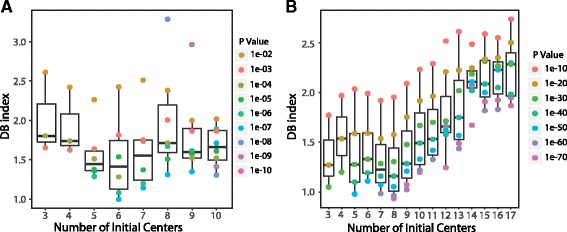
Distribution of DB index values calculated by SAIC algorithm for lung epithelial dataset and cell mixture data set. **a** DB indexes were calculated for each *p* value and initial center k combination after the SAIC algorithm converged using the 80 epithelial lung single cell dataset. Boxplot of DB indexes of different *p* values are shown for each initial center. Each dots represents the DB index value for each *p* value. **b** Similarly, DB indexes were calculated using the 301 single cell mixture data set

The first biological dataset we tested had RNA-seq data of 80 single lung epithelial cells. Genes with FPKM value more than 1 in at least two cells (*N* = 10,421) were used in the loess model fitting and filtered as described in the method session. After the filtering steps, 4272 genes were selected for the subsequent signature gene selection. We tested initial center K from 3 to 10 because the optimal number of clusters is 5 based on the original published paper, and *P* value ranging from 0.001 and 1e-10 since lower *p* values would not yield any signature genes. A DB index matrix was generated based on the exhaustive search with all combinations of *p*-value and cluster number within the search space and presented in the boxplot graph (Fig. 3a). Similar to the results of simulation data, the DB index is the worst for initial center of 3. The DB index distribution is better when the chosen initial center increases and approaches 6, but become worse for 7 centers and above. Out of the 72 combinations, the best DB index value appears at K = 6 and *P* = 1e-07. As a result of this parameter combination, 216 genes were identified as the signature gene set.

**Fig. 4:**
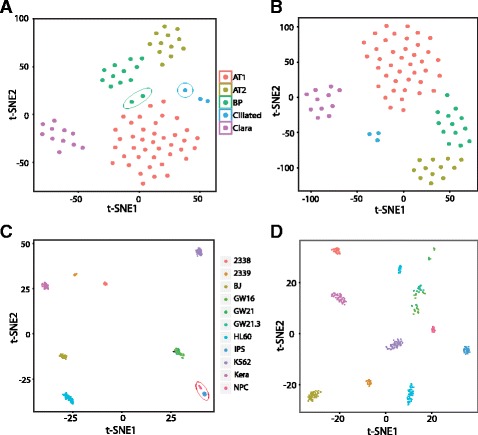
Comparison of SAIC with other methods using t-SNE plots. Two dimensional t-SNE plots showing clustering of 80 lung epithelia cells into five cell groups, using (**a**) 216 signature genes identified by SAIC, and (**b**) 111 genes identified by PCA. Clusters are colored by cell class as reported in the paper published by Treutlein et al. The cells that are clustered differently from the published results are circled. The two BP cells are circled in green, and the one ciliated cell is circled in blue. The t-SNE plots of clustering results of 301 mixed single cells are shown in C and C. they are based on (**c**) 410 signature genes selected by SAIC and (**d**) 1545 variable genes selected by Seurat clustering algorithm. Color represents different cell sources correspondent to the paper published by Pollen et al. The iPS and NPC cells are circled in red

**Fig. 5:**
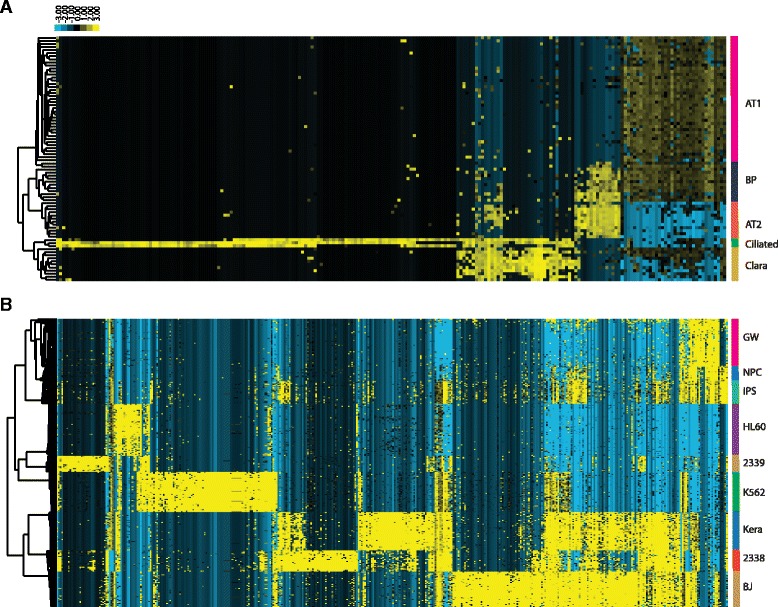
Gene expression heatmap based on hierarchical clustering of (**a**) 80 lung epithelia cells, using 216 signature genes identified by SAIC and (**b**) 301 mixed single cells, using 410 signature genes selected by SAIC. Side color bar was labeled based on literature reported cell group information

As shown in Fig. 4a, cells can be clustered into 6 groups using the 216 signature genes identified by our method. Similar to the clustering result of PCA-method selected genes (Fig. 4b), the SAIC algorithm plot shows that the location of BP cell cluster is between the AT1 and AT2 clusters, consistent with the fact that BP cells express genes found in both AT1 and AT2 cells. However, two of the originally designated BP cells are classified as AT1 cells in our analysis. From the t-SNE plot, these two cells are indeed closer to AT1 cell clusters (Fig. 4a) and show different expression profiles than the other BP cells using a heatmap (Fig. 5a). Ciliated cells and Clara cells form distinct clusters by both SAIC and PCA methods (Fig. 4a and b), except that one ciliated cell forms a separate cluster as its expression profile is slightly different from the other two ciliated cells (Figs. 4a and 5a). To compare the clustering results using the signature genes selected using our approach and the ones selected in the original paper using the PCA method, we calculated the DB index score. We found that our clustering result had a better DB index (1.18) than the PCA-relied clustering result (1.25), suggesting the signature genes selected by our approach had clustered cell groups better than the PCA method. Hierarchical clustering of the 80 cells using the 216 gene set also shows clearly distinct cell groups that are consistent with the t-SNE plot (Fig. 5a).

### Cell mixture data

This dataset contains RNA-seq data of 301 cells, which is a mixture of 11 different cell types. Three cell types, GW16, Gw21, AND GW21.2, are very similar to each other, as they are cells at different developmental stages. After initial filtering steps similar to the lung single cell data above, 7591 genes remained for subsequent signature gene selection. The search space of *p*-values and initial center numbers were selected differently from the previous data set due to the different scale of sample size and diversity of the cell sources. We wanted to examine whether more groups would give better results, and also whether our algorithm had bias towards bigger group numbers, so we extended the number of initial centers to 17 as there were more cells in the data set. We also used much smaller *P* value cutoffs as the cells are quite distinct based on their expression profiles.

As shown in Fig. 3a, initial center K = 8 gives the best overall DB index, and the trend of DB index distribution is similar to the other two data sets, as it gets better when approaching the optimal initial center. The combination of K = 8 and *P* = 1e-70 gives the best DB index value and results in 410 signature genes. The number of signature genes is much smaller compared to the 1545 genes selected using Seurat’s default method. Fig. 4c shows that the 301 cells can be clustered into 8 distinct groups, representing the different cell types. The three human cortex cells at different development stages (GW16, GW21, and GW21.3) are grouped into a single cluster, reflecting the nature of their similar sources. The NPC and iPS cells are close to each other and grouped into a single cluster, reflecting that they share similar progenitor cell characteristics (Fig. 4c). The groups are more separated from each other on the t-SNE plot generated from our signature genes and have less dispersion within groups, comparing to the Seurat clustering approach (Fig. 4c and d). Our approach also has a better DB index score (1.73 vs. 1.82), indicating our signature genes can separate different cells groups better than the Seurat approach. The heatmap derived from hierarchical clustering using the same set of signature genes also separates the cells into groups correspondent to the expected cell population, with iPS and NPC cells clustered together (Fig. 5b).

To investigate whether our signature genes are biological meaningful and could provide some useful information about cell types in each cluster, we did further analysis on the genes uniquely identified in each cell cluster to see whether we could identify their possible tissue type. Using DAVID online annotation tools [21, 22], the 135 genes specifically detected in K562/HL60/2339 cells are enriched in genes that originated from blood cells (Table 1A). Similarly, the 29 genes specifically detected in NPC/GW/iPS clusters and 236 genes in BJ/Kera/2338 cluster indeed originate from neural cells and skin cells (Table 1B and C). These results confirm that our signature genes not only are useful to cluster the cells, but also are biologically informative.

**Table 1:** DAVID functional annotations of tissue expressions by genes specified by A) blood cell clusters; B) dermal cell clusters; C) neural cell clusters

Term	*P*Value
A) blood cell clusters	
B-cell	4.82E-08
Blood	2.83E-07
Whole blood	7.46E-07
Bone marrow	7.16E-05
Peripheral blood	8.28E-05
Cord blood	0.001507
B) dermal cell clusters	
Pancreas	4.00E-12
Placenta	8.29E-09
Keratinocyte	1.25E-08
Liver	1.36E-08
Fibroblast	6.40E-06
Epidermis	1.41E-04
C) neural cell clusters	
Fetal brain cortex	5.31E-07
Cajal-Retzius cell	8.08E-06
Fetal brain	0.0050
Eye	0.015
Epithelium	0.029
Muscle	0.029

## Discussion

The rapid advances in single cell transcriptomics empower scientists to explore gene expression at single cell resolution, which increases the demand for developments of single cell data analysis approaches. In this study, we developed an iterative algorithm called SAIC that combines k-means clustering and ANOVA analysis, using the exhaustive search within the search space to select the signature genes that will form optimal clustering of single cells. As a result of this method, the identified signature gene set has a much more manageable size to investigate the underlying biological meaning.

SAIC is robust on both simulated and real datasets. Using the simulated data set, we have demonstrated that our algorithm could accurately detect the pre-defined signature genes and cell clusters. For both of the real single cell datasets, SAIC could effectively separate the cells into very distinct groups and had better performance than the Seurat/PCA clustering approach based on DB index evaluation.

The performance of the algorithm is also good. With the large dataset of 301 single cells, the running time was only a few hours with modest powered computation server. For the smaller Lung data set with 80 cells, analysis was finished in just under 30 min. Most importantly, SAIC is less arbitrary than the PCA based method with regard to parameter setting. When using the PCA based method, a user has to arbitrarily decide which principle components to include, and how many genes with the highest loading in each PC will be considered. Changing those parameters can result in very different gene lists and clustering patterns. For example, the authors of the lung epithelial dataset combined the top 30 genes in each of the first 4 PCs. This selection was probably based on examination of clustering results from various parameter combinations and human intervention to decide the optimal parameters. In our method, there are also two parameters, initial center K and *P* value for the ANOVA test. While the results are depending on the initial selection of these two parameters, when a reasonable search space is selected, the algorithm will be able to identify the best combination based on DB index score. For initial center number K, prior biological knowledge can provide a reasonable range in most cases. The DB index distribution can also be used as guidance for the parameter selections, as one should observe the lowering trend when the optimal number of center is being approached (Fig. 2). If this trend is not observed in the selected range, one needs to change the number of initial centers K to identify a better search space. *P*-value range is usually affected by the extent of differences among the cell groups. For instance, in the cell mixture data, the lower bound *P* value was 1e-70 because of the large differences among different cell types. This lower bound *P* value was identified by lowering the *P* value stepwise until no genes could be identified beyond that level of significance. Therefore, a reasonable search space can be defined quickly with minimum initial trials.

The SAIC algorithm also has limitations. For instance, it may not detect very rare cell populations that contain less than 3 cells, as they may not reach the desired significance level during an ANOVA test and hence could be missed in the iteration process. We used log2 RPKM values for the two biological data sets tested in this study. It has been reported that quantifying the transcript compatibility counts (TCC) is better than RPKM/FPKM [23]. It has been shown that cell cycle-related genes may account for false positive groups in single cell RNA-seq data [2]. We did not try to remove these genes in the current implementation, but one can remove these genes using the algorithm proposed by Buettner et al. [2], then applying the SAIC algorithm. In addition, SAIC still relies on the initial selection of two parameters to identify the best combination, although the optimal search space can be identified easily with some initial trials.

## Conclusions

In this study, we have presented a novel algorithm for single-cell RNA-seq analysis that identifies the optimal set of signature genes to separate single cells into distinct groups. We have shown that its performance is superior to PCA based method using both simulated data and published single cell RNA-seq data.

## Additional file

Additional file 1: Figure S1. Two dimensional t-SNE plots showing clustering results of 100 cells in the simulation dataset using PCA (A) and Seurat (B) method. For PCA method, we combined the top 50 genes of the first 4 principal components to select 347 unique genes. For Seurat, we picked the first 3 principal component and significant level of 0.01, which resulted in 183 unique genes. These data were used to generate t-SNE plots using Seurat package (PDF 262 kb)
