# Supplementary figures and images for: SAIC: an iterative clustering approach for analysis of single cell RNA-seq data
Source: BMC Genomics. 2017 Oct 3;18(Suppl 6):689. doi: 10.1186/s12864-017-4019-5 (PMC5629617; doi:10.1186/s12864-017-4019-5)

Suppl. Figure 1

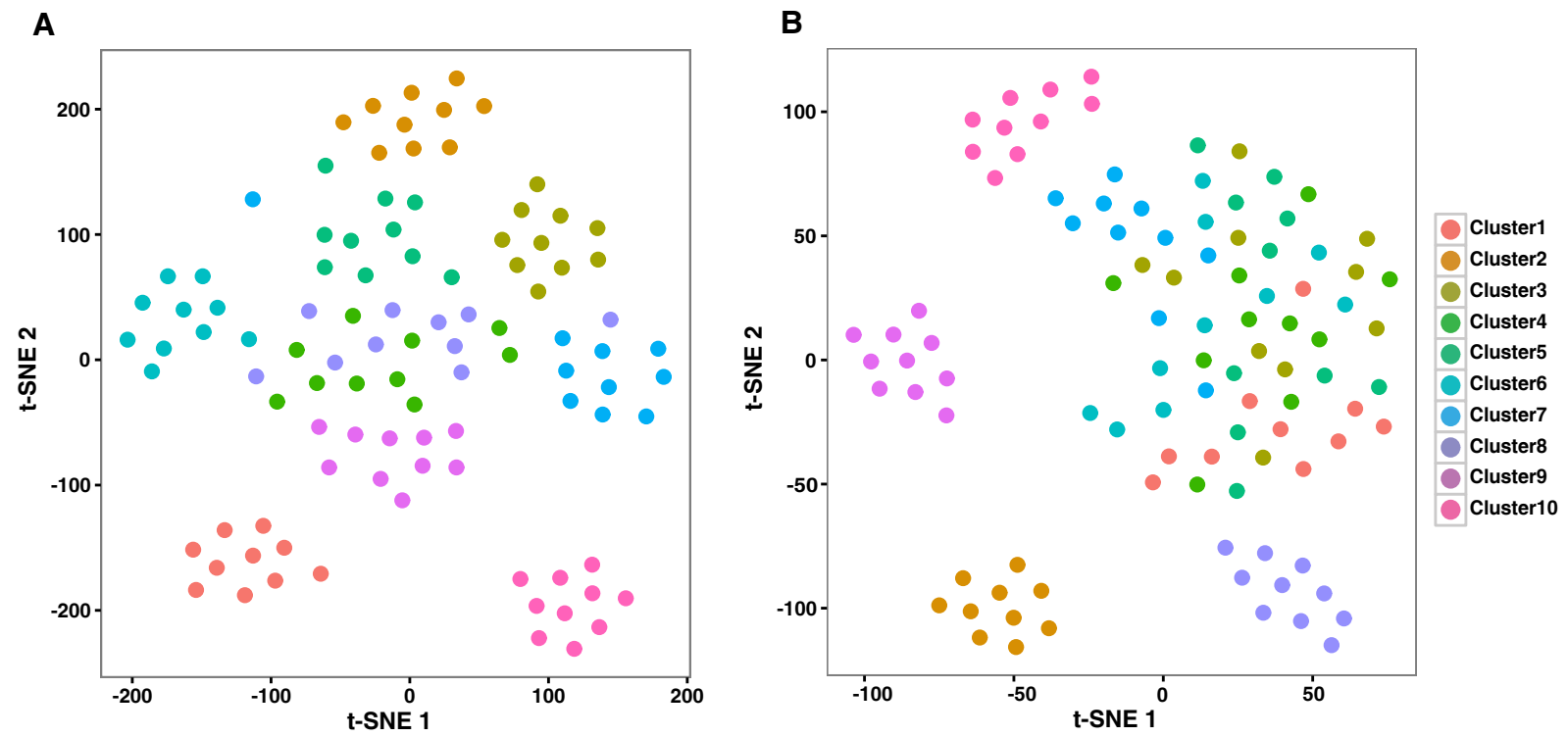

Supplement: Additional file 1: Figure S1. — Two dimensional t-SNE plots showing clustering results of 100 cells in the simulation dataset using PCA (A) and Seurat (B) method. For PCA method, we combined the top 50 genes of the first 4 principal components to select 347 unique genes. For Seurat, we picked the first 3 principal component and significant level of 0.01, which resulted in 183 unique genes. These data were used to generate t-SNE plots using Seurat package (PDF 262 kb) [file 12864_2017_4019_MOESM1_ESM.pdf]
